# Supplementary figures and images for: The mammalian target of rapamycin (mTOR) kinase mediates haloperidol-induced cataleptic behavior
Source: Transl Psychiatry. 2020 Oct 2;10:336. doi: 10.1038/s41398-020-01014-x (PMC7532208; doi:10.1038/s41398-020-01014-x)

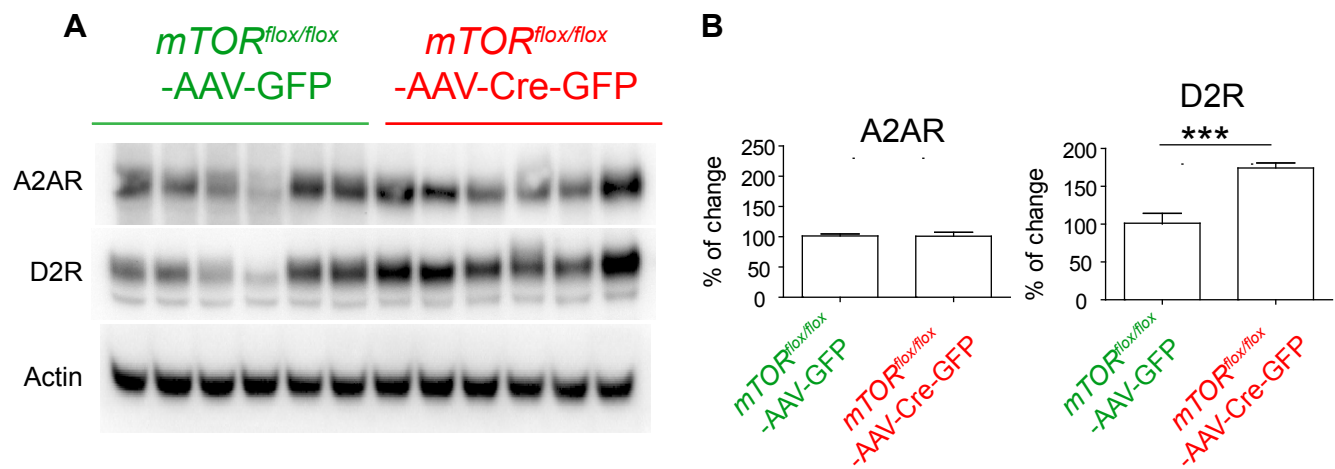

Supplementary Figure 1

Supplement: Supplementary file 1 — Suppementary Figure 1 [file 41398_2020_1014_MOESM1_ESM.pdf]
